# Myeloid-Related Protein Activity in Rheumatoid Arthritis

**DOI:** 10.4061/2011/580295

**Published:** 2011-08-15

**Authors:** Charles J. Malemud

**Affiliations:** Division of Rheumatic Diseases, Department of Medicine, University Hospitals Case Medical Center, Case Western Reserve University School of Medicine, 2061 Cornell Road, Suite 207, Cleveland, OH 44106, USA

## Abstract

SA100A8, SA100A9, and SA100A12 are members of the myeloid-related protein class. SA100A8 and SA100A9, also known as MRP-8 and MRP-14, respectively, are intracellular Ca^2+^-binding proteins produced mainly by neutrophils and monocytes where they exist as a heterodimeric complex in the cytosol. The MRP-8/-14 complex has been shown to promote chronic inflammation associated with rheumatoid arthritis (RA). In that regard, MRP-8 and MRP-14 regulate the inflammatory response through their capacity to recruit neutrophils and monocytes to target tissues resulting in attachment to endothelium. MRPs also activate the signal transduction pathway principally involving the stress-activated/mitogen-activated protein kinases. MRP-8/MRP-14 also increased nitric oxide synthesis. Most recently, the MRP-8/MRP-14 complex was shown to be a novel ligand for the toll-like receptors (TLRs) and TLR-4, in particular. Engagement of TLRs by the MRP-8/-14 complex may be particularly important for activating antigen-presenting dendritic cells which regulate critical autoimmune responses that promote chronic synovitis characteristic of RA.

## 1. Introduction

 The myeloid-related protein (MRP) family of proteins include MRP-8, also known as S100A8, MRP-14, also known as SA100A9, and S100A12 [1]. MRP-8 and MRP-14 are intracellular Ca^2+^-binding proteins that are produced by a variety of myeloid cells. MRP-8 and MRP-14 exist as a heterodimeric complex in the cytosol of polymorphonuclear leukocytes and monocytes [2, 3]. 

## 2. The MRP Complex and Inflammation 

 MRPs have been implicated as important contributors to inflammation in general, and to the overall inflammatory response associated with autoimmune disorders such as rheumatoid arthritis (RA) [4, 5]. The MRP-8/14 complex, in particular, has been proposed to play a critical role in regulating several of the inflammatory responses associated with RA because both MRP-8 and MRP-14 can promote chronic inflammation and act to recruit neutrophils and monocytes to inflamed tissue by enhancing their migration, retention, and attachment to the endothelium [6]. In this regard, the best evidence for the fundamental role played by the MRPs in inflammation [7, 8] can be gleaned from the results of experimental studies which demonstrated that exogenously added MRP-8/14 was capable of directly inducing macrophage recruitment to inflamed tissues which was also accompanied by increased levels of nitric oxide (NO) [8, 9]. 

## 3. The MRP Complex, NO, and Signal Transduction

NO has been shown to be an important soluble mediator of inflammatory responses in adult RA via the upregulation of inducible nitric oxide synthase (iNOS) as a result of nuclear factor *κ*B (NF-*κ*B) activation [10]. In addition, NO has been shown to be a potent inducer of apoptosis in cultured articular chondrocytes [11, 12]. Importantly, MRP-8/14-mediated up-regulation of iNOS gene expression by macrophages *in vitro* was also shown to also be accompanied by the activation (i.e., phosphorylation) of multiple protein kinase-mediated signal transduction pathways, including those involving c-Jun-N-amino-terminal kinase (JNK), extracellular-regulated kinase 1/2 (ERK1/2), and Janus kinase/signal transducers and activators of transcription (JAK/STAT) as well as activation of NF-*κ*B. Interestingly, JAK/STAT was found to be preferentially activated by interferon-*γ* (IFN-*γ*) rather than by MRP-8/14 [4]. However, the activation of multiple downstream signal transduction pathways by NO, IFN-*γ*, MRP-8/14, and other proinflammatory cytokines, namely, tumor necrosis factor-*α* (TNF-*α*), IL-6, IL-8, IL-12/23, IL-18, and so forth [13] has profound implications for any future therapeutic interventions for RA [14–17] because it would be unlikely that suppression or inhibition of any one of these signaling pathways would ameliorate the inflammation of RA or reduce articular cartilage destruction and/or subchondral bone erosions characteristic of RA [18].

## 4. Tissue Localization and Circulating MRP Complex in RA

 MRP antigens were found to reside only in the macrophage subtype in the synovial membrane of RA synovial tissue [4]. Importantly, the relevance of MRP antigen localization to synovial joint cartilage destruction and subchondral bone erosion in RA was also supported by the finding that MRP antigens were localized only to those cells that were adjacent to the cartilage-pannus junction and only in RA patients with clinically active disease [4]. These findings suggested the possibility that suppression of MRP gene expression might be a suitable novel target for therapeutic intervention to inhibit the progression of juvenile chronic arthritis [19, 20] and/or adult RA [8]. 

 Importantly, in RA patients, the serum levels of MRP-8/14 and SA100A12 correlated with one another [5]. Moreover, serum levels of MRP-8/14 and S100A12 in 138 patients with RA were substantially elevated compared to 44 healthy controls. The S100A9 serum levels were detected using an anti-MRP-14 antibody that not only measured MRP-14 but also detected MRP-14 in its complex with MRP-8. These levels were about 100-fold lower than those reported in other studies [21, 22], and the differences between the results of these two studies [5, 21] could be accounted for on the basis of the specificity of the anticalprotectin (an antibody generated against the MRP-8/14 complex) that was previously employed [21, 22]. Taken together, these results indicated that elevated levels of circulating serum MRP antigens in RA patients were coordinately increased. Thus, the level of circulating MRP-8/14/SA100A12 could potentially be employed as a biomarker to track the pathologic and radiographic progression of RA as well as clinical responses and other objective measurements to approved RA therapeutic DMARDs and/or biologic agents. 

 In addition to the above, elevated levels of these MRP antigens were also shown to significantly contribute to vascular injury and thus to the peripheral end-organ damage in adult RA [5] which both significantly contribute to the numerous comorbid components of RA disease activity. The elevated levels of peripheral and organ-related MRP antigens may also be associated with precocious atheroma formation and the accelerated development of atherosclerosis which has been found to correlate with vascular injury and the later development of coronary artery disease [23, 24] which is often seen as a significant comorbidity in RA patients with clinically active disease [25–27]. Interestingly, carotid intima media thickness (cIMT), a marker of preclinical atherosclerosis, and flow-mediated dilatation (FMD) of the brachial artery, a marker of endothelial dysfunction, were assessed in 79 patients with early RA and in 44 age-matched and sex-matched controls [28]. Importantly, the FMD and cIMT at baseline were not significantly different between the two groups. Thus, the results of this study indicated that although preclinical evidence of atherosclerosis was often associated with early RA, there was also a strong indication that early therapeutic intervention in RA (perhaps by targeting MRP-8/14 antigens) could establish a means to significantly reduce the progression of atherosclerosis in these RA patients. 

 Importantly, in systemic-onset juvenile RA (JRA), a strong and positive correlative relationship was found between the level of MRP-8/14 in the peripheral circulation and the level of another proinflammatory cytokine, germane to RA disease activity and progression, namely, interleukin-1*β* (IL-1*β*), [29]. This result suggested that in JRA, the MRP-8/14 complex in conjunction with IL-1*β* constituted a novel positive feedback pathway that could account for the robustness of macrophage activation in this arthritic disorder of childhood.

## 5. What Is the Evidence That the MRPs Help Drive RA Disease Progression?

### 5.1. Toll-Like Receptors (TLRs)

TLR-mediated signaling is a crucial component in driving both innate and adaptive immune activation [30]. Recently, the MRP-8/14 complex was shown to be a novel ligand for the TLR pathway and for TLR-4, in particular [31, 32]. Thus, in a fashion similar to LPS, a known activator of TLR-mediated signaling, MRP-8 induced the hyperphosphorylation of IL-1 receptor-associated kinase-1 in human IFN-*γ*-primed monocytes that was also characterized by elevated levels of TNF-*α*, IL-1*β*, and IL-12 [32]. In addition, LPS and MRP-8 were used to determine the extent to which these responses occurred in murine phagocytes without a functional TLR-4 complex. Thus, compared to their wild-type counterparts, TLR-4 mutant mice demonstrated a similar lack of response to both LPS and MRP-8, but in contrast to wild-type mice, stimulation of TLR-9, -8, or -7 with cpG and R848, respectively, revealed no differences in response. These data indicated that MRP-8 was an endogenous ligand of TLR-4 and that the MRP-8/-14 complex stimulation of TLR-4 was a mechanism for increasing the effect of LPS stimulated by the MRP-8/14 complex. In this regard, the TLR pathway having been engaged by the MRP-8/14 complex may be particularly important for activating antigen-presenting dendritic cells which regulate, in part, several of the autoinflammatory and autoimmune responses, which in the context of RA promotes the chronic state of synovitis [32].

### 5.2. Chemokines and Adhesion Molecules

 The elevated production of chemokines and adhesion molecules are also crucial to the pathogenesis of experimentally-induced murine arthritis [33] and human RA [34]. Thus, TNF-*α* not only promotes the inflammatory responses associated with RA [34] but also enhances the migration of neutrophils towards CCL3, known as macrophage inhibitory protein-1*α* (MIP-1*α*), via its capacity to interact with CCR5 [34, 35]. 

 The MIP-1*α*/CCR5 response also appears to be a consequence of TNF-*α*-induced up-regulation of CD11b/CD18, also known as macrophage-activating complex-1, (Mac-1), which was found on the surface of neutrophils. In this regard, Newton and Hogg [36] found that MRP-14 activated Mac-1 on the surface of neutrophils, whereas Rouleau et al. [37] showed that SA100A12 induced enhanced neutrophil migration and adhesion as well as release from mouse bone marrow at concentrations comparable to those levels of SA100A12 found in the plasma of patients with inflammatory arthritis. Therefore, it was noteworthy that BIIL3, a long-acting leukotriene B4 (LTB4) receptor antagonist, inhibited LTB4-induced Mac-1 expression in patients with RA [38].

### 5.3. MRP Levels in Response to Antirheumatic Disease Therapies

Although a rapid lowering of MRP levels in synovial tissue sublining macrophages was associated with the positive clinical response of RA patients to anti-TNF-*α* therapy [39, 40], could MRP levels be employed as a sensitive biomarker for distinguishing between effective and ineffective drug interventions? In this regard, the data from 2 randomized, controlled RA clinical trials were re-examined in which experimental therapies designed to neutralize either monocyte chemotactic protein-1 (MCP-1) activity or the interaction between C5a and its receptor C5aR were assessed with each of these strategies failing to show clinical efficacy. In this analysis, Wijbrandts et al. [41] combined the data from these 2 studies with clinical response and CD68 data from other RA clinical trials in which combinations of DMARDs and biological agents resulted in a positive clinical response. The “standardized response mean” (SRM) was employed as the outcomes measure. The results of this study showed a strong relationship between lower disease activity score-28 (DAS-28) levels (a standard for measuring clinical response in RA clinical trials) and the number of CD68-positive sublining macrophages (SRM, >0.8). This result indicated that the number of CD68-positive sublining macrophages which had been previously shown to be positive for MRP [39] was a sensitive biomarker to measure clinical responses to effective therapies in RA clinical trials, but not for the interventions which were ineffective (anti-MCP-1, SRM, 0.19; anti-C5aR, SRM, -0.071). Furthermore, the difference in the weighted mean of the SRM between clinically effective and ineffective interventions was significant for both DAS-28 and the number of CD68 sublining macrophages which indicated that the weighted mean of the SRM could be used to detect differences between effective and ineffective interventions as well as placebo effects [41]. It should be noted that Haringman et al. [42] had also previously shown a similar strong correlation between lowered DAS-28 scores and the number of CD68 sublining macrophages (Pearson correlation, 0.874, P < 0.01) in response to several types of antirheumatic therapies. 

 Additionally, patients with spondyloarthropathy or adult RA who responded favorably to the anti-TNF-*α* monoclonal antibody infliximab showed a rapid and marked decrease in the level of MRP-positive macrophages in tissue infiltrates, but not in the total population of resident macrophages [43]. Furthermore, infliximab effectively reduced the soluble form of MRP-8/14 in the sera of RA patients but had no effect on the level of the soluble form of CD163 [39]. 

 It has also been important to assess the extent to which the number of MRP-positive macrophages in RA patients in response to anti-TNF-*α* therapy correlated with changes in other biomarkers of inflammation. To address this issue, van Kuijk et al. [44] showed that lower DAS-28 scores could be achieved in patients with psoriatic arthritis receiving the anti-TNF-*α* monoclonal antibody, adalimumab compared to placebo. In this analysis of psoriatic arthritis, patients who favorably responded to adalimumab significantly reduced levels of matrix metalloproteinase-3 (MMP-3) and MMP-13, and a trend towards lower levels of von Willerbrand factor, IL-1 and IL-6, was found. Moreover, clinical improvement in these psoriatic arthritis patients in response to adalimumab was strongly correlated with reduced numbers of CD3/CD4-positive T cells, and MRP-8-positive macrophages. 

 Taken together, these results suggested that suppression of Mac-1 expression or blockade of TNF-*α*-mediated cellular signaling by anti-TNF-*α* therapies may result in the inhibition of migration and adhesion of neutrophils and macrophages into inflamed tissue, in part, by regulating MRP complex-mediated migration and adhesion of these inflammatory cells to specific end-organ sites. Thus, measurements of MRP-8 was one of several biomarkers, including MMP-3, MMP-13, and CD68 (a marker for macrophages expressing MRP-8) but not CD163 (a marker for resident tissue macrophages) in the sublining of synovial tissue as well as neutrophils. In effect, changes in the levels of these biomarkers were shown to be useful in making predictions regarding the clinical responses of patients with spondyloarthropathy to infliximab [45] but were somewhat in conflict with the results reported by De Rycke et al. [43] who determined that anti-TNF-*α* therapy appeared to alter the expression of cell-specific CD163 as well as CD68.

### 5.4. Regulation of Signal Transduction Pathways: Cell Survival and Apoptosis

Signal transduction pathways that are activated in RA in response to elevated levels of proinflammatory cytokines [13, 18, 46] include JNK, ERK 1/2, p38 kinase, and JAK/STAT which may all be differentially activated by the MRP complex [4]. In that regard, Morel et al. [47] recently demonstrated that JNK activity was essential for regulating the phosphorylation of Mcl-1 (a member of the Bcl2-related protein family) by glycogen synthase kinase 3 (GSK3). Importantly, activation of Mcl-1 may also be responsible for controlling the survival and/or apoptosis of neutrophils, macrophages, and other cells essential in activating the inflammation cascade in RA. Thus, in future studies, it will be of interest to determine the extent to which the MRP protein complex regulates the activation of Mcl-1 and GSK3. 

 Finally, MRP-14 was shown to be a potent *activator* of p38 kinase-dependent functional responses in intact human neutrophils that included the regulation of neutrophil survival and apoptosis [48]. Thus, suppression of MRP-14 activity may be important in the context of developing novel therapeutic strategies for regulating neutrophil-associated survival in RA via suppression of MRP complex-mediated signaling.
